# Identification of drought-responsive and novel *Populus trichocarpa* microRNAs by high-throughput sequencing and their targets using degradome analysis

**DOI:** 10.1186/1471-2164-14-233

**Published:** 2013-04-09

**Authors:** Peng Shuai, Dan Liang, Zhoujia Zhang, Weilun Yin, Xinli Xia

**Affiliations:** 1 College of Biological Sciences and Biotechnology, National Engineering Laboratory of Tree Breeding, Beijing Forestry University, Beijing 100083, P.R. China

**Keywords:** *Populus trichocarpa*, microRNA, Drought, Target identification

## Abstract

**Background:**

MicroRNAs (miRNAs) are endogenous small RNAs (sRNAs) with a wide range of regulatory functions in plant development and stress responses. Although miRNAs associated with plant drought stress tolerance have been studied, the use of high-throughput sequencing can provide a much deeper understanding of miRNAs. Drought is a common stress that limits the growth of plants. To obtain more insight into the role of miRNAs in drought stress, Illumina sequencing of *Populus trichocarpa* sRNAs was implemented.

**Results:**

Two sRNA libraries were constructed by sequencing data of control and drought stress treatments of poplar leaves. In total, 207 *P*. *trichocarpa* conserved miRNAs were detected from the two sRNA libraries. In addition, 274 potential candidate miRNAs were found; among them, 65 candidates with star sequences were chosen as novel miRNAs. The expression of nine conserved miRNA and three novel miRNAs showed notable changes in response to drought stress. This was also confirmed by quantitative real time polymerase chain reaction experiments. To confirm the targets of miRNAs experimentally, two degradome libraries from the two treatments were constructed. According to degradome sequencing results, 53 and 19 genes were identified as targets of conserved and new miRNAs, respectively. Functional analysis of these miRNA targets indicated that they are involved in important activities such as the regulation of transcription factors, the stress response, and lipid metabolism.

**Conclusions:**

We discovered five upregulated miRNAs and seven downregulated miRNAs in response to drought stress. A total of 72 related target genes were detected by degradome sequencing. These findings reveal important information about the regulation mechanism of miRNAs in *P*. *trichocarpa* and promote the understanding of miRNA functions during the drought response.

## Background

MicroRNAs (miRNAs) are one of the most abundant classes of small RNAs (sRNAs) in plants and animals. These endogenous sRNAs were first identified in a metazoan called *Caenorhabditis elegans* in 1994 [1] and were subsequently identified in plants [2] and viruses [3]. MiRNAs are typically 21 nucleotides (nt) in length and play regulatory roles at the post-transcriptional level by repressing translation or directly degrading target message RNAs (mRNAs) [4]. Plant miRNA genes are first transcribed into primary miRNAs, and then processed into miRNA precursors with stem-loop structures by Dicer-like proteins. Finally, they are released into the cytoplasm by cleavage into an miRNA::miRNA* duplex from the nucleus [5]. The mature miRNAs join an RNA-induced silencing complex (RISC), and the RISC targets specific mRNAs and downregulates the expression of target mRNAs [6]. MiRNAs participate in various processes such as metabolism [7], growth [8], development [9,10], biotic [11] and abiotic [12-19] stress tolerance.

An increasing body of evidence indicates that miRNAs are involved in the plant drought stress response [13-15,17,20,21]. In *Arabidopsis*, four drought-responsive miRNAs (miR396, miR168, miR167, and miR171) have been identified by microarray analysis [12]. In tobacco, nine miRNAs strongly induced by drought stress have been experimentally identified, among which miR395 and miR169 are the two miRNAs most sensitive to drought stress [14]. In rice, 30 miRNAs have been identified as significantly down- or upregulated under drought stress using a microarray platform [13]. In *Medicago truncatula* (*M*. *truncatula*), Wang et al. (2011) mined drought-responsive miRNAs on a genome-wide scale using the Illumina sequencing technology; 22 members from four miRNA families and 10 members of six miRNA families were identified as up- and downregulated in response to drought, respectively [17]. Li et al. (2011) reported 104 upregulated and 27 downregulated miRNAs by Illumina sequencing and microarray profiling in *Populus euphratica* (*P. euphratica*) [15]. Furthermore, Qin et al. (2011) confirmed three upregulated and two downregulated mature miRNAs in response to drought using a RT-qPCR assay [16].

Environmental stressors due to climate change, especially drought stress, could make forests increasingly vulnerable to disease and die-offs [22]. Drought may have a profound effect on forest health [23]. With its modest genome size and rapid, widespread growth, *P*. *trichocarpa* was the first model forest species sequenced [24]. Lu et al. (2005) studied miRNAs in *P*. *trichocarpa* and identified stress-responsive and novel miRNAs by Sanger sequencing technology [25]. An additional 15 novel *P*. *trichocarpa* miRNAs were further identified by Klevebring et al. (2009) using the 454 sequencing method [26]. Further study is needed to elucidate the mechanism of regulation of *P*. *trichocarpa* miRNA in general and of drought-responsive miRNAs in particular.

Only 234 *P*. *trichocarpa* miRNA precursors are annotated in the miRBase (version 18.0) [27], compared to 581 and 635 for *Oryza sativa* and *M*. *truncatula*, respectively, two other model organisms. Since the genome size of *P*. *trichocarpa* (423 Mbp, JGI version 3.0) is similar to that of *M*. *truncatula* (approximately 454–526 Mbp) and rice (389 Mbp), the potential for identification of new, specific miRNAs in *P*. *trichocarpa* is great. In this context, high-throughput sequencing was used to identify non-conserved miRNAs and drought-responsive miRNAs with the new version of the poplar genome (version 2.0), which has not been used in previous research on *P*. *trichocarpa*. The targets of these conserved and novel miRNAs were predicted, and some of them were confirmed by degradome sequencing. We discussed the potential regulatory mechanism between miRNAs and their targets. This may help to unravel the mechanism of drought stress tolerance in *P*. *trichocarpa* and other plants.

## Results

### Illumina sequencing of *P*. *trichocarpa* leaves under control and drought conditions

According to a previous study on the relative soil moisture content (RSMC) of water-deficient soil [15,28], *P*. *trichocarpa* plants were subjected to control levels (RSMC, 70–75%) and drought levels (RSMC, 15–20%). The two libraries were sequenced by an Illumina sequencer, yielding 27,333,282 reads for the control library (CL) and 30,806,496 reads for the drought library (DL) (Additional file 1: S1). After removing the low-quality sequences and adapter sequences, 26,229,957 clean sequences in CL and 30,233,516 clean sequences in DL were obtained, comprising 2,730,022 and 2,834,584 unique sequences, respectively (Table 1).

**Table 1:** **Sequencing of miRNAs in *Populus *plants**

***Populus *plants**	**Tissues**	**Techniques**	**Redundant reads (x10000)**	**Unique reads (x10000)**	**Reference**
*P.trichocarpa*	stem	Sanger	-	-	[29]
*P.trichocarpa*	leaf and stem	Sanger	-	-	[25]
*P.balsamifera*	leaf	454-FLX	4.13	0.60	[30]
	bud		3.56	0.63	
*P.trichocarpa*	leaf	454-FLX	90.19	8.05	[26]
*P.euphratica*	leaf(control)	Illumina	703.51	-	[15]
	leaf(treatment)		818.66	-	
*P.beijingensis*	stem(control)	Illumina	1827.39	115.70	[11]
	stem(treatment)		1818.23	141.72	
*P.trichocarpa*	leaf(control)	Illumina	2623.00	160.92	-
	leaf (treatment)		3023.35	179.28	

**Figure 1:**
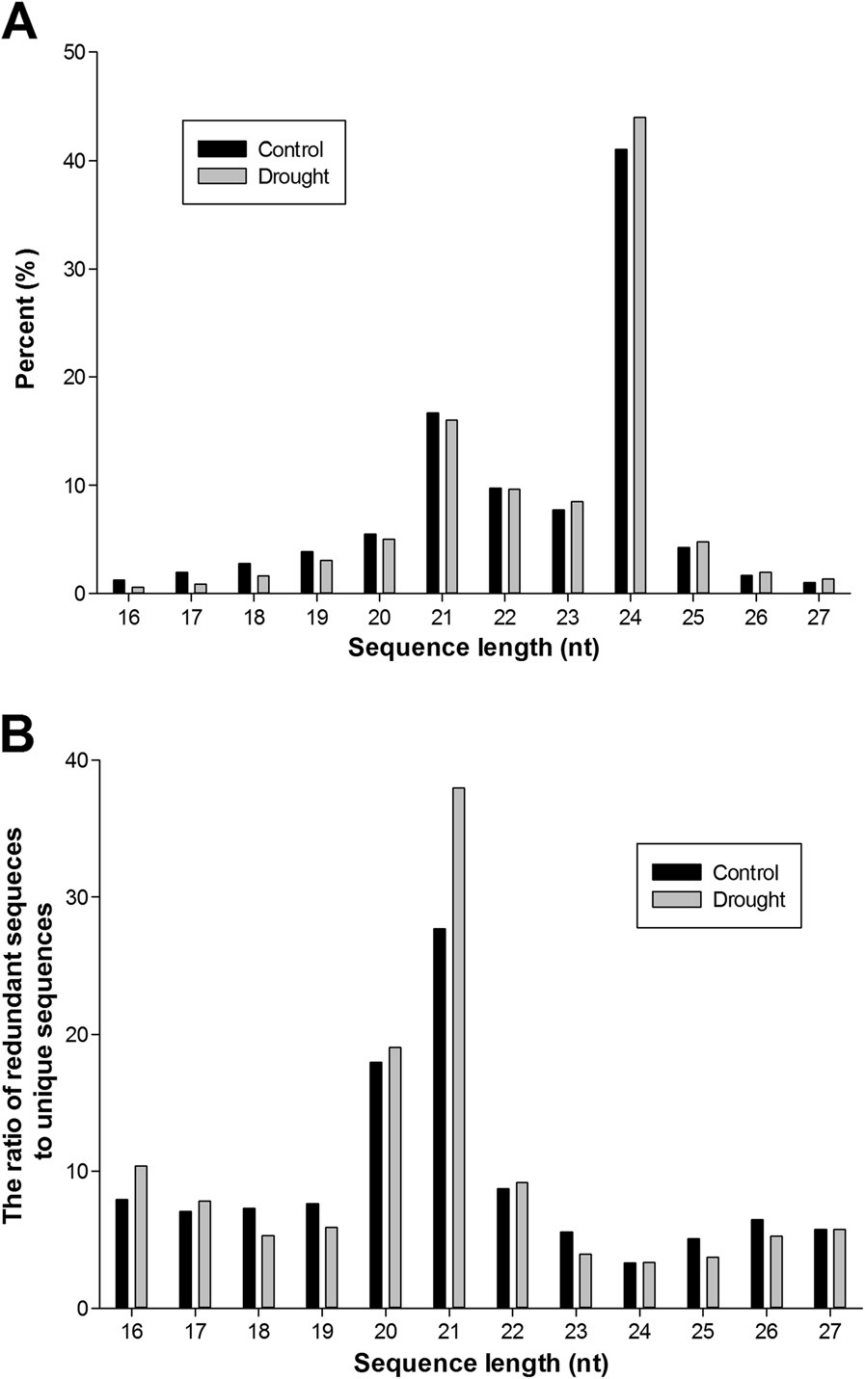
**Sequence length distribution of *P. trichocarpa *sRNAs.** The size distribution of all unique sRNAs of the two libraries is show in panel **A**. The ratio of redundant and unique sequences of sRNAs of the two libraries is show in panel **B**.

The size distribution of all unique sRNAs is summarized in Figure 1A. The displayed length of *P*. *trichocarpa* sRNA ranged from 16 to 27 nt, and the two major size classes were 24 nt (41.06% in CL and 43.94% in DL) and 21 nt (16.64% in CL and 16.05% in DL). This is in agreement with previous studies on sRNAs of *P*. *trichocarpa *[26] and *M. truncatula *[17] using high-throughput sequencing. To analyze the average abundance of each length between sRNAs of CL and DL further, we measured the ratio of raw and unique reads (Figure 1B). The redundancies of sRNAs varied widely in length, and the 20 and 21 nt sRNAs displayed the highest redundancies. The average ratio of redundant and unique sequences of sRNAs of the two libraries showed obvious changes in 21 nt sRNAs; the redundancy of DL was 37.16% greater than that of CL. This may be why drought stress strongly induced the expression of these 21 nt sRNAs; most conserved miRNAs belong to this group.

After genomic annotation of the *P*. *trichocarpa* sRNAs, small interfering RNA (siRNA) and miRNA with various important post-transcription regulating functions were the largest of our acquired sequences. The siRNA is a 22 to 24 nt double-strand RNA, each strand of which is 2 nt longer than the other on the 3’ end [31]. These aligned sequences might represent siRNA candidates. In total, deep sequencing obtained 577,393 and 956,979 siRNA candidates after the control and drought stress treatments, respectively (Additional file 1: file S1). Interestingly, the ratio of siRNA reads to all sRNAs reads increased sharply from 2.20% (CL) to 3.17% (DL). To obtain the annotation of known miRNAs, sRNAs were aligned to the miRBase 18.0 of *P*. *trichocarpa*. In total, 10,784,410 and 15,674,365 sequencing reads were identified as known poplar miRNAs in the two libraries. Thirty-four families from 207 known miRNAs were found, which accounted for about 87.3% of the total members. The remaining 30 miRNAs were not detected (Additional file 2: S2), possibly because of the tissue specificity of expression in poplar.

### Novel non-conserved miRNAs from *P. trichocarpa*

After identifying potential siRNAs and conserved miRNAs from the unique sRNA sequences, the remaining sRNA sequences were potential candidate miRNAs. For the identification of new miRNA, the primary criterion was a stable hairpin structure. After pooling the reads of the two libraries and analyzing the precursors of potential miRNAs using the MFOLD web server, we found 274 potential candidate miRNAs (Additional file 3: S3 and Table 2). In compliance with the plant miRNA criteria of Meyers [32], only 65 miRNAs with star sequences from 54 families were chosen as novel non-conserved miRNAs. Of these, 47 miRNAs were 21 nt long, eight miRNAs were 22 nt, five miRNAs were 20 nt and the remaining five were 19 nt long. The nucleotide bias at the first nucleotide showed a tendency to be U (41% of 65 novel non-conserved miRNAs). This would allow for easier miRNA RISC loading assisted by AGO protein [33] and is consistent with the trend of conserved miRNAs in plants [34].

**Table 2:** **New miRNAs in *P. trichocarpa***

		**Counts of**					**Found**
**miRNAs**	**Sequences(5’-3’)**	**miRNAs**	**miRNA Locations**	**Arm**	**LP**	**MEF**	**by**
		(miRNAs*)			**(nt)**	**(kcal/mol)**	**others**
Ptc-miRn1	CUGUUAUGAAUUGAUGGAGUG	71(10)	scaffold_14:13399032:13399142:-	3’	111	−56.3	
Ptc-miRn2	UGGUAAUGCAAGUGUUGCUAA	22(15)	scaffold_11:1025164:1025342:+	3’	179	−67.2	
Ptc-miRn3	UAGAUUGUUUUUAUGCUUUGA	19(2)	scaffold_16:10809578:10809788:+	5’	211	−106.5	D
Ptc-miRn4	CUCUUCAAAUAAAUCGUGGGA	380(12)	scaffold_4:10873191:10873312:-	3’	122	−66.8	
Ptc-miRn5	AAUGUUGUUAUUAACACUGUA	127(27)	scaffold_4:20884831:20884933:+	3’	103	−48.2	
Ptc-miRn6a	UUAUGCAUUUUUGUCCCUCGC	171(1)	scaffold_4:2221707:2221834:+	3’	128	−64.5	
Ptc-miRn6b	UUAUGCAUUUUUGUCCCUCGC	171(52)	scaffold_5:24265933:24266061:+	3’	129	−63.2	F
Ptc-miRn6c	UUAUGCAUUUUUGUCCCUCGC	171(49)	scaffold_5:24270121:24270264:+	3’	144	−67.7	
Ptc-miRn6d	UUAUGCAUUUUUGUCCCUCGC	171(1)	scaffold_5:24280115:24280237:+	3’	123	−51.6	F
Ptc-miRn7a	UCUUAUGCGUUUUUGUCUCU	238(3)	scaffold_5:24266243:24266355:+	3’	113	−60.7	D
Ptc-miRn7b	UCUUAUGCGUUUUUGUCUCU	238(26)	scaffold_5:24270440:24270552:+	3’	113	−61.6	CD
Ptc-miRn8	UUUGGUUAUUGUCUCGAGACA	1664(23)	scaffold_15:1581044:1581277:-	5’	234	−95.6	
Ptc-miRn9	UUUCUUAUCGAUCACUAGACG	41(1)	scaffold_1112:3373:3393:+	3’	116	−54.4	
Ptc-miRn10	UCUCUUCUGUUCCUGAACGGU	16(7)	scaffold_1:15618173:15618365:+	3’	193	−63.4	
Ptc-miRn11	UUGCUGAAACGAUUGAACUAU	70(2)	scaffold_9:10835006:10835091:+	3’	86	−48.6	E
Ptc-miRn12	UCUUGAGAACAUGAUGAAUCG	11(1)	scaffold_11:4410758:4410914:+	5’	157	−57.7	
Ptc-miRn13	UGAUGAUUAAUUGACUGCAAA	754(51)	scaffold_1:34025510:34025606:+	5’	97	−60.1	E
Ptc-miRn14	AUCAUAUAGGUUGAUCCUCGU	18(1)	scaffold_6:6189111:6189190:-	5’	80	−58.6	
Ptc-miRn15	UCUGUCGCUGGAAAGAUGGUAC	57000(57)	scaffold_17:10902829:10902928:+	5’	100	−66.7	AE
Ptc-miRn16	AGAUGGGCAUCGGCAUUGUGA	116(7)	scaffold_13:797593:797704:-	3’	112	−48.9	
Ptc-miRn17	CGCUCGCCAGCGUUGCACCACC	618(247)	scaffold_1:10455697:10455799:-	5’	103	−47.3	E
Ptc-miRn18	UUUGAAAUUGAACAAAUGGUA	10(1)	scaffold_5:17113917:17114027:+	5’	111	−64.6	
Ptc-miRn19	UUGCAUGCAUGAACUUGAAAU	178(4)	scaffold_4:20393549:20393757:+	3’	209	−81.1	B
Ptc-miRn20	UUGAGAAAAGUCAAUCGGACC	11(1)	scaffold_13:7256997:7257111:-	5’	115	−48.5	
Ptc-miRn21	UGUUCAGAUCAGUAGAUAGCA	1310(29)	scaffold_8:207741:207864:-	5’	124	−46.6	BE
Ptc-miRn22	UAGAGCAGAUUGUAAGGGAAG	528793(209)	scaffold_1:5198865:5198974:-	3’	110	−49.3	CDE
Ptc-miRn23	UUGAAGAAAGGUAGACAGAUAG	207(1)	scaffold_3:14589106:14589377:-	3’	272	−74.3	D
Ptc-miRn24a	CGAACGUUGACCGAAUGUGAA	15(3)	scaffold_10:11417598:11417684:-	5’	87	−31.9	
Ptc-miRn24b	CGAACGUUGACCGAAUGUGAA	15(3)	scaffold_1646:4003:4023:+	5’	88	−35.4	
Ptc-miRn25	UUGUACACAGAAUAGGUGAAAU	1624(7)	scaffold_5:1237647:1237753:+	3’	107	−35.4	CE
Ptc-miRn26	UUACCAAGUUUCAAAUUCUCA	8(5)	scaffold_16:2500220:2500318:-	5’	99	−53.1	
Ptc-miRn27	GCUAGGACCAAGUUUUUUGGA	367(50)	scaffold_13:9970810:9970906:+	5’	97	−58	
Ptc-miRn28	GAUGACAUGGACACCAAAAUC	9(6)	scaffold_1:35822741:35822925:-	5’	185	−64	
Ptc-miRn29	ACACAGAAACUCCAAGCCCAC	48(2)	scaffold_15:14059579:14059742:-	3’	164	−88.4	
Ptc-miRn30	UGAUCUAGAGAACCGUUGCU	38(3)	scaffold_15:5977842:5977957:+	5’	116	−54.5	
Ptc-miRn31	UACAUGUAGAGACCACCAAAC	75(51)	scaffold_10:2022789:2023023:+	5’	235	−97.1	
Ptc-miRn32	UGUCGCAGGAGAGAUGGCGCUA	216(21)	scaffold_17:10888890:10889033:+	5’	144	−67.8	D
Ptc-miRn33	UAGUUCCCAACCUACACCACA	6(2)	scaffold_12:5445549:5445646:+	5’	98	−63.8	
Ptc-miRn34	UAAUUAGAACUCAUACUAGAC	44(4)	scaffold_13:3364782:3364868:+	5’	87	−38.6	
Ptc-miRn35	UUGCCGACCCCACCCAUGCCAA	1127(309)	scaffold_10:12814698:12814812:-	3’	115	−49	D
Ptc-miRn36	UGGAUGAUCAUGUUGGCAACC	1614(364)	scaffold_4:6132662:6132818:+	3’	157	−60.3	BE
Ptc-miRn37	UGUGAUAAUGGAGGCUAUGGU	88(1)	scaffold_1:5941972:5942066:+	3’	95	−75.8	E
Ptc-miRn38	UAAUAAAAUCUCGACUAUUAU	249(4)	scaffold_11:535912:536007:-	5’	96	−44	
Ptc-miRn39	UGGACUCCUUUGGGGAGAUGG	216(2)	scaffold_15:12480557:12480647:+	3’	91	−41.1	BE
Ptc-miRn40	UCGAAUUUGGGCUUGAGAUUG	50787(2158)	scaffold_3:9383285:9383395:+	3’	111	−45.4	ABE
Ptc-miRn41	GGAAACCUUUUGUGGGGGUUU	491(9)	scaffold_15:12329506:12329600:-	3’	95	−44.7	DE
Ptc-miRn42	GGCAUGAGGUGUUUGGCAAGA	1721(1155)	scaffold_5:11901572:11901665:-	5’	94	−38.9	A
Ptc-miRn43	GAUGGGAUUUUUCGGGAAGUG	41(13)	scaffold_10:17756662:17756763:+	3’	102	−52.3	
Ptc-miRn44	GUUUUCCCUGAAUCACUCCCA	15(7)	scaffold_5:21920822:21920920:-	5’	99	−55.8	
Ptc-miRn45a	UGGGUGGGAGGUGUGGUAGCU	8(1)	scaffold_5:9237322:9237483:-	5’	162	−79.9	
Ptc-miRn45b	UGGGUGGGAGGUGUGGUAGCU	8(1)	scaffold_724:8193:8213:+	5’	166	−78.7	
Ptc-miRn46	ACAUGUGCUGGGUAGGAGGAA	21(4)	scaffold_6:9390506:9390758:-	3’	253	−86.8	
Ptc-miRn47	AGUGGCAUUGGAGGUAUCCC	297(18)	scaffold_1:22901250:22901371:-	3’	122	−34.7	D
Ptc-miRn48	UCUCAAGCCCAAAUUCGAUC	8(2)	scaffold_3:9383296:9383382:-	5’	87	−35	B
Ptc-miRn49	CAAGGAGUAAUUAGUGACAUC	974(1)	scaffold_9:7737213:7737443:+	5’	231	−63.3	
Ptc-miRn50	UGAUAUGUGGCAUUCAAUCGA	2880(8)	scaffold_3:14915775:14915873:+	3’	99	−77.1	E
Ptc-miRn51a	GCCGAGCUCGGGGAUUGCG	84645(465)	scaffold_17:2344664:2344909:+	3’	246	−141.5	
Ptc-miRn51b	GCCGAGCUCGGGGAUUGCG	84645(465)	scaffold_17:2357226:2357471:+	3’	246	−143	
Ptc-miRn51c	GCCGAGCUCGGGGAUUGCG	84645(465)	scaffold_17:2307047:2307292:+	3’	246	−137.4	
Ptc-miRn51d	GCCGAGCUCGGGGAUUGCG	84645(465)	scaffold_17:2279166:2279411:+	3’	246	−137.6	
Ptc-miRn51e	GCCGAGCUCGGGGAUUGCG	84645(465)	scaffold_17:2268545:2268790:+	3’	246	−139.3	
Ptc-miRn52	GGGGGUUGCUGUCAAGCAUAA	61(19)	scaffold_18:2804678:2804833:+	3’	156	−74.6	B
Ptc-miRn53	UAAAUCAAGCCCGGUACUUUU	10(2)	scaffold_11:15209261:15209374:-	5’	114	−42.6	
Ptc-miRn54a	UUCAUUCCUCUUCCUAAAAUGG	1827(506)	scaffold_15:8556706:8556831:+	5’	126	−61	ABCE
Ptc-miRn54b	UUCAUUCCUCUUCCUAAAAUGG	1827(500)	scaffold_12:7706162:7706287:-	5’	126	−50.9	ABCDE

‘LM’, Length of miRNAs. ‘nt’, neuleotides. ‘LP’, Length of precursors. ‘A’, refer to [26]; ‘B’, refer to [11]; ‘C’, refer to [19]; ‘D’, refer to [15]; ‘E’, refer to [10]; ‘F’, refer to [18].

**Figure 2:**
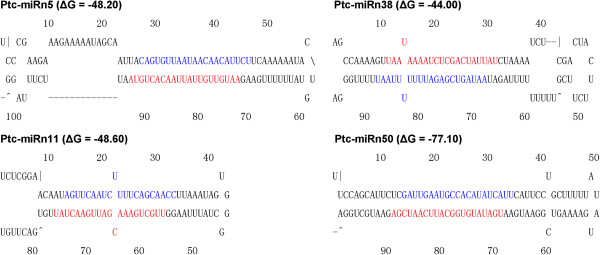
**Predicted miRNA precursor’ hairpin structures of new miRNA precursors.** Precursor structures of 4 newly identified poplar miRNAs (miRn5, miRn11, miRn38, and miRn50) were predicted by MFOLD pipeline. The MEFs were list after the miRNAs name. The mature miRNA and miRNA star sequences are highlighted in red and blue, respectively.

These RNA structures were predicted by MFOLD software and manually checked according to the criteria of Meyers [32]. The lowest minimum free energy (MFE) of all hairpin structures of the novel miRNAs precursors was −31.9 kcal/mol (Table 2), which is slightly lower than the threshold of −30 kcal/mol reported in a previous study [35]. All precursors of novel miRNAs had regular hairpin structures (Additional file 4: S4), and four of these (Ptc-miRn5, Ptc-miRn11, Ptc-miRn38, and Ptc-miRn50) are presented in Figure 2.

### Differential expression of miRNAs in *P*. *trichocarpa*

**Figure 3:**
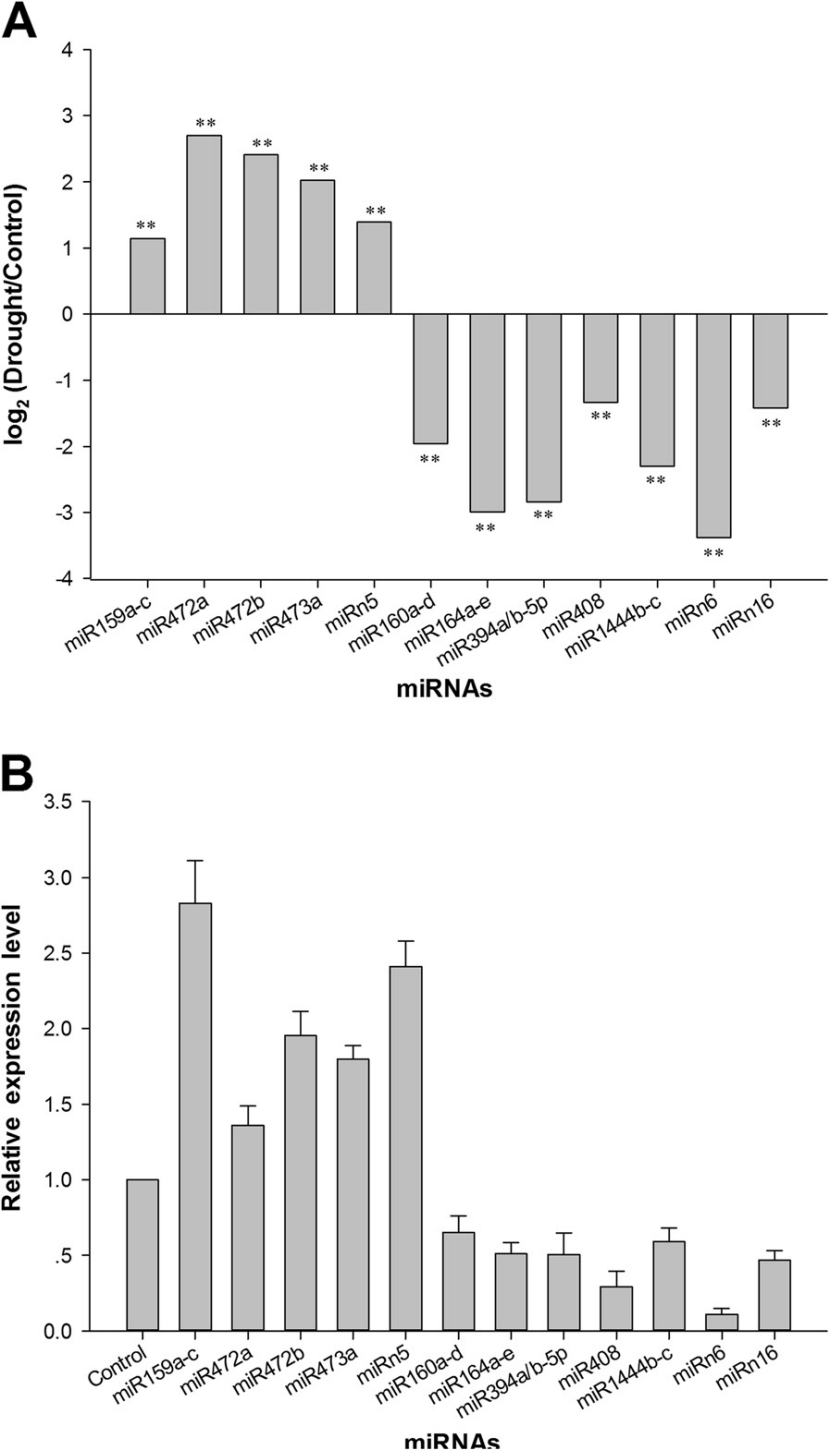
**Differential expression analysis of conserved and novel drought-responsive miRNAs.** The changes in miRNAs for CL and DL are greater than 2-fold. For each miRNA, sequence reads were divided by the total sequence number then multiplied to 1,000,000 (reads per million). Differential expression of known and new miRNAs in response to drought stress by sequencing is shown in panel **A**. The positive and negative values mean miRNAs whose expression was stimulated and suppressed by drought stress, respectively. ** mean significant difference between control and drought stress at P ≤ 0.01. The relative expression level of miRNAs measured by RT-qPCR in response to drought stress is shown in panel **B**.

To identify drought-responsive miRNAs from *P*. *trichocarpa*, the number of normalized miRNA reads of CL and DL were compared. Based on the sequencing results, the differential expression of miRNAs greater than two-fold were chosen for experimental validation by quantitative real time polymerase chain reaction (RT-qPCR) (Additional file 5: S5). As shown in Figure 3, the expression patterns of the sequencing and RT-qPCR results of drought-responsive miRNAs were consistent, both indicating that four miRNAs (Ptc-miR159a-c, Ptc-miR472a, Ptc-miR472b, and Ptc-miR473a) were upregulated after drought treatment, and that five miRNAs (Ptc-miR160a-d, Ptc-miR164a-e, Ptc-miR394a/b-5p, Ptc-miR408, and Ptc-miR1444b-c) were downregulated by drought stress [25].

We further analyzed the expressions of the 65 new miRNAs under the two treatments. The drought-responsive miRNAs are listed in Figure 3; all were confirmed by the sequencing and RT-qPCR results. Among the 65 miRNAs, two novel miRNAs (Ptc-miRn6a-d and Ptc-miRn16) were downregulated by drought stress, and only miRn5 was upregulated in response to drought stress (Additional file 5: S5).

### Target analysis of novel and conserved miRNAs by degradome sequencing

The previously known miRNA targets also identified in this study are available on the PopGenIE site (http://bioinformatics.cau.edu.cn/PMRD/adjunct/ptc_miR_target.txt). For new miRNAs whose targets were not known, we predicted their targets using the plant target prediction pipeline by the *P. trichocarpa* genome V2.0. The rules used for target prediction were based on those suggested by Allen et al. (2005) [36] and Schwab et al. (2005), as follows: (i) no more than four mismatches between the sRNA and the target (G-U bases count as 0.5 mismatches); (ii) no more than two adjacent mismatches in the miRNA/target duplex; (iii) no adjacent mismatches in positions 2–12 of the miRNA/target duplex (5’ of miRNA); (iv) no mismatches in positions 10–11 of the miRNA/target duplex; (v) no more than 2.5 mismatches in positions 1–12 of the miRNA/target duplex (5’ of miRNA); and (vi) the minimum free energy (MFE) of the miRNA/target duplex should be equal or greater than 74% of the MFE of the miRNA bound to its perfect complement [37]. We predicted 281 targets for 53 miRNA families; the other six were not found (Additional file 6: S6).

The verification of miRNA targets would provide further evidence for the existence of new non-conserved miRNAs. To identify the miRNA targets, two parallel analyses of RNA ends (PARE) libraries were constructed for the *P*. *trichocarpa* degradome sequencing. In particular, the sRNA-cleaved mRNAs ligated by 5^′^ RNA adapters used for degradome sequencing acquired 23,326,117 and 34,398,368 reads (longer than 18 nt) in the mRNA libraries of the two treatments after removing redundancy; 108,593 and 234,316 unique reads could be matched to the *P*. *trichocarpa* genome (version 2.0) without mismatches (Additional file 7: S7) [38]. Fifty-three conserved and 19 new miRNA-targeted transcript pairs were confirmed by degradome sequencing. The target transcripts were pooled and categorized into three classes with reference to *Arabidopsis*[39]. Eleven pairs of miRNAs and their targets belonged to category I, which accounts for the most abundant sequence reads at the cleavage site. A total of 8 and 53 miRNAs and transcript pairs belonged to categories II and III, respectively. In addition, 13 target transcripts were predicted previously by either PopGenIE site or us (Table 3).

**Table 3:** **Targets of *P. trichocarpa *miRNAs verified by degradome sequencing**

**miRNA**	**Target transcript**	**Category**	**Cleavage**	**Reads at**	**Position**	**Target annotation**
			**Site**	**Cleavage**	**Penalty**	
				**Site**	**Score**	
Ptc-miR156k	POPTR_0013s06910.1	III	799	224	4	Glucose-6-phosphate/phosphate
Ptc-miR159a-c	POPTR_0011s11490.1	III	428	153	4	Methionine sulfoxide reductase B 1
Ptc-miR159e	POPTR_0006s16050.1	III	1014	134	4	Catalytic
Ptc-miR159f	**POPTR_0002s05060.1**	III	372	99	4	Protein kinase
Ptc-miR164f	POPTR_0007s12070.1	III	623	337	4	PS II oxygen-evolving complex 1
Ptc-miR164f	POPTR_0005s13860.1	III	623	337	4	PS II oxygen-evolving complex 1
Ptc-miR166a-q	**POPTR_0009s01990.1/2**	I	574	469	3	Transcription factor
Ptc-miR166a-q	**POPTR_0004s22090.1**	I	563	766	2.5	Transcription factor
Ptc-miR167a-e	POPTR_0012s09730.1	I	946	694	3.5	NOP56 (Arabidopsis homolog of
						nucleolar protein)
Ptc-miR167f/g	POPTR_0013s01580.1	II	285	239	4	Function unknown
Ptc-miR169z	POPTR_0018s11310.1	III	2204	118	4	Eukaryotic translation initiation factor 4F
Ptc-miR171l-n	**POPTR_0003s06200.1**	III	1615	89	3	Function unknown
Ptc-miR172a-c/f	POPTR_0014s06330.1	III	799	110	4	Glycoprotease M22 family
Ptc-miR172d/e	POPTR_0016s02570.1	III	103/172	1222	4	Function unknown
Ptc-miR172d/e	**POPTR_0001s44680.1**	III	186	111	4	Apolipoprotein D-related
Ptc-miR172g/h	**POPTR_0001s44680.1**	III	186	111	3.5	Apolipoprotein D-related
Ptc-miR172i	POPTR_0015s13920.1	III	446	174	3.5	Chlororespiration reduction 1
Ptc-miR390a-d	POPTR_0017s07350.1	II	2472	689	3.5	LOX2 (lipoxygenase 2)
Ptc-miR390a-d	POPTR_0001s05330.1	II	2622	689	3.5	LOX2 (lipoxygenase 2)
Ptc-miR390a-d	POPTR_0005s11640.1	III	9458	108	4	FAT domain-containing protein
Ptc-miR390a-d	POPTR_0328s00200.1	II	2622	1180	3.5	LOX2 (lipoxygenase 2)
Ptc-miR396a/b	POPTR_0004s18020.1/2	III	547	140	3.5	MYB4
Ptc-miR396f	POPTR_0004s18020.1/2	III	547	140	3.5	MYB4
Ptc-miR397c	POPTR_0002s01740.1	III	135	163	3.5	PETE1 (plastocyanin 1)
Ptc-miR397c	POPTR_0009s05960.1	III	1660	104	4	Hydrolase
Ptc-miR398a	**POPTR_0010s06990.1/2**	III	1428	346	4	DC1 domain-containing
Ptc-miR473a	POPTR_0602s00210.1	I	850	327	4	VEP1 (vein patterning 1)
Ptc-miR475d	POPTR_0003s20310.1/3	I	1898	544	4	Zinc finger (CCCH-type) family protein
Ptc-miR476a	POPTR_0001s21140.1	III	145	137	3.5	Post-illumination chlorophyll
						Fluorescence increase protein
Ptc-miR403a-c	**POPTR_0016s03150.1**	III	637	156	4	Cytochrome p450
Ptc-miR403a-c	**POPTR_0016s03160.1**	III	634	156	4	Cytochrome p450
Ptc-miR482.1	POPTR_0017s06100.1	I	1247	1312	3.5	UDP-glucosyl transferase
Ptc-miR482.1	POPTR_0009s13280.1	III	1036	94	4	Translation initiation factor
Ptc-miR482.1	POPTR_0010s13860.1	III	152	101	4	ATP-dependent Clp protease
						Adaptor protein
Ptc-miR482.1	POPTR_0017s06080.1	I	914	1312	3.5	UDP-glucosyl transferase
Ptc-miR482.1	POPTR_0322s00200.1	I	1247	1312	3.5	UDP-glucosyl transferase
Ptc-miR482.1	POPTR_0017s06120.1	I	1010	1312	3.5	UDP-glucosyl transferase
Ptc-miR482.1	POPTR_0001s27260.1	III	1033	144	3.5	Carotenoid cleavage dioxygenase 1
Ptc-miR482.1	POPTR_0009s06530.1	III	1051	144	4	Carotenoid cleavage dioxygenase 1
Ptc-miR482.1	POPTR_0007s00550.1	III	337	228	4	NADP-dependent
Ptc-miR530b	POPTR_0002s24070.1	III	424	104	4	Photosystem I subunit L
Ptc-miR1444a	POPTR_0014s10790.1	III	2569	159	3.5	Preprotein translocase secA
Ptc-miR1444a	POPTR_0014s11380.1	III	2368	135	3.5	Pentatricopeptide (PPR)
Ptc-miR1446a-e	POPTR_0014s06190.1/2	II	677	511	4	Unfertilized embryo sac 10
Ptc-miR1446a-e	POPTR_0001s34380.1/2	III	295	178	4	16S rRNA processing
Ptc-miR1446a-e	POPTR_0011s13770.2	III	55	204	3.5	UBQ10 (Polyubiquitin 10)
Ptc-miRn6	**POPTR_0001s11990.1/2/3**	III	259	98	1	Function unknown
Ptc-miRn7	**POPTR_0001s11990.1/2/3**	III	259	98	0.5	Function unknown
Ptc-miRn7	POPTR_0014s01740.1/2	II	723	366	3.5	APT1 (Adenine phosphoribosyl
						transferase 1)
Ptc-miRn8	POPTR_0001s40480.1	III	656	1443	4	Glycolate oxidase
Ptc-miRn13	POPTR_0018s08940.1	III	1605	116	4	Abnormal inflorescence meristem
Ptc-miRn26	**POPTR_0016s00510.1**	III	1233	265	4	Heat shock protein 81-4
Ptc-miRn30	POPTR_0002s07540.1	III	528	75	3.5	SKP2A (F-box protein)
Ptc-miRn32	POPTR_0014s16800.1	III	504	93	4	Beta-catenin repeat family protein
Ptc-miRn33	POPTR_0019s08960.1	III	3244	134	4	Function unknown
Ptc-miRn37	POPTR_0003s08800.1	III	585	108	4	Pentatricopeptide (PPR)
Ptc-miRn38	POPTR_0011s17280.1	III	584	170	4	PEX5 (Peroxin 5)
						Signal-1 binding
Ptc-miRn40	**POPTR_0001s07220.1**	III	305	79	2	mTERF(mitochondrial transcription
						termination factor family protein)
Ptc-miRn40	POPTR_0005s20140.1	III	1030	119	4	Thylakoidal ascorbate peroxidase
Ptc-miRn49	POPTR_0011s01280.1	III	791	90	4	Copper/zinc-superoxide
						dismutase 2

‘Cleavage site’, the cleavage site location at the area of coding sequence; ‘Position penalty score’, the same penalty score as the prediction of new miRNA targets; ‘MFE’, minimum free energy of the stem loop structure. The bold font of transcripts indicates that they were identified by prediction.

Plant miRNAs have a strong propensity for target genes with important functions [34]. According to the biological functions described by UniProt (http://www.uniprot.org/), these target transcripts can be grouped into nine categories. The majority of targets fall into the stress-response category, suggesting that these genes are drought-responsive (Table 4). Several other groups contain genes that regulate transcription, oxidative reduction, transport, and lipid metabolism. In this study, miR396 targeted a MYB transcription factor, and Ptc-miRn30 targeted an F-box family protein. The annotation of targets not only indicated some transcription factors and F-box proteins, but also some superoxide dismutases (SODs) and other proteins involved in glucose and lipid metabolism. A Cu-Zn SOD was targeted by Ptc-miRn49. All of these results indicate that miRNAs and their targets are reliable.

**Table 4:** Function category of the identified target transcripts

**Gene function**	**Number of targets**
Stress response	18
Oxidation reduction	8
Metabolism	6
Transcription	5
Lipid metabolism	4
Regulation of transcription	1
Transporter	1
Proteolysis	1
Other	15
Total	59

## Discussion

### High-throughput sequencing of *Populus*

In a comparison of six *Populus* miRNA studies (Table 1) [11,15,25,26,29,30], two used traditional Sanger sequencing [25,29], two others used 454-pyrosequencing [26,30], and the remaining two used the latest Illumina sequencing technology (as in the present study) [11,15]. Along with the rapid development of sequencing technology, CL and DL can result in more sequences and greater sequencing depths than those reported in previous publications, due to the high throughput of the Illumina sequencer. In our study, because of the in-depth search, a large number of novel non-conserved miRNAs were found. The *P*. *trichocarpa* genome of Version 2.0 was used in this study; the transcript assemblies of the *P*. *trichocarpa* genome Version 2.0 are more meticulous than those of Version 1.1. This can increase the likelihood of finding more new miRNAs in general and drought-induced novel miRNAs in particular.

### Novel miRNAs

Compared to six previous studies of *Populus* plants [10,11,15,18,19,26], we identified 28 novel miRNAs have been identified (Table 2). Eleven of these were found at least once. On comparing the miRNA counts, 24 had counts greater than 100. Interestingly, two of the members of the Ptc-miRn54 family are the most frequently and robustly miRNAs identified in poplar high-throughput sequencing studies. Furthermore, the counterparts of Ptc-miRn40, Ptc-miRn52, Ptc-miRn54a, and Ptc-miRn54b in *P*. *beijingensis* were verified by RT-qPCR [11]. This provides more, strong evidence for the novel miRNAs identified from *P*. *trichocarpa*.

### Drought-responsive miRNAs in *P*. *trichocarpa*

Although miRNAs have been shown to play an important role in the drought stress response of *P*. *trichocarpa*[25], little information on high-throughput sequencing of *P*. *trichocarpa* is available in this area. The present study on drought-responsive miRNAs from *P*. *trichocarpa* will improve the understanding of the drought response of this species. We identified nine conserved miRNAs and three novel miRNAs that show significant changes in response to drought stress. The results were confirmed by both high-throughput sequencing and RT-qPCR. To obtain more information, we compared the identified drought-responsive miRNAs with those identified in other studies (Table 5) [12-15,17,21,25,40-46]. MiR159 and miR164 have not yet found to be drought-responsive in *Populus* plants, except in this research. In addition, miR472, miR473, and miR1444 were found to be drought-responsive only in *Populus* plants, including in this study. The regulatory direction of four miRNAs (miR160, miR472, miR473 and miR408) was identical in *P. tomentosa* and our research, which might be due to their close genetic relationship.

**Table 5:** MiRNAs responsive to drought stress in diverse plant species

**miRNA(↑&↓)**	**Drought in other publication(↑&↓)**	**Refs**
miR159(↑)	*Arabidopsis thaliana*(↑), *Nicotiana tabacum*(↓), *Oryza sativa*(↓), *Panicum. Virgatum*(↑)	[40-43]
miR 160(↓)	*Populus tomentosa*(↑&↓), *Saccharum* spp. (↑)	[21,44]
miR 164(↓)	*Medicago truncatula*(↓), *S*. spp. (↑&↓)	[17,44]
miR 394(↓)	*Glycine max*(↑), *P. tomentosa*(↑), *S*. spp. (↓)	[21,44,45]
miR 472(↑)	*P. tomentosa*(↑)	[21]
miR 473(↑)	*Populus euphratica*(↓),*P. tomentosa*(↑)	[15,21]
miR 408(↓)	*A. thaliana*(↑), *Hordeum vulgare*(↑), *M. truncatula*(↑), *O. sativa*(↓), *P. tomentosa*(↓)	[12,13,21,46]
miR 1444(↓)	*Populus trichocarpa*(↓)	[25]

‘↑’, upregulated; ‘↓’, downregulated; ‘↑&↓’, some members were upregulated, some members were downregulated.

We further studied the target genes of these drought-responsive miRNAs by sequencing of the degradome library and comparing our work to previous studies [25,29]. We found two upregulated miRNAs (Ptc-miR472 and Ptc-miRn5) that were both predicted to target putative disease resistance proteins in *P*. *trichocarpa* (Additional file 5: S5) [25]. The cross adaptation between disease resistance and drought stress tolerance in plants exists through unknown mechanisms. Ptc-miR159 is another upregulated miRNA; its *Arabidopsis* homolog targets an MYB transcription factor. The ABA-induced accumulation of the miR159 homolog makes the MYB transcript degradation desensitize hormone signaling during seedling stress responses in *Arabidopsis *[40]. According to our degradome sequencing results, the Ptc-miR159 was confirmed to target a methionine sulfoxide reductase (MSR). The homologs of MSR were induced by biotic and abiotic stresses in plants [47-50]. They catalyze the reduction of methionine sulfoxide to methionine [47] and play a major role in regulating the accumulation of reactive oxygen species (ROS), which can damage proteins in plant cells [50]. Regulation of the MSR gene by Ptc-miR159 may occur through a homeostatic mechanism in response to drought stress in *P*. *trichocarpa*.

Ptc-miR473 was also upregulated in drought stress. It targets a member of a plant-specific GRAS transcription factor gene family [29]. Another member of this family (PeSCL7) from *P*. *euphratica* was confirmed to play key roles in salt and drought stress tolerance [51]. In the present study, Ptc-miR473 was confirmed to be targeted to Vein Patterning 1 (VEP1), which belongs to a short-chain dehydrogenase/reductase (SDR) superfamily [52]. The homolog of VEP1 in *Arabidopsis* was confirmed to be required for vascular strand development and to be upregulated by osmotic stress [52,53]. Ptc-miR473 regulates the expression the GRAS protein and VEP1, both of which were responsive to drought stress, this may be the drought tolerance mechanism in *P*. *trichocarpa*.

The number of downregulated miRNAs was larger than the number of upregulated miRNAs. The two downregulated miRNAs (miR160 and miR164) were both identified to be cold-responsive miRNAs in *P*. *trichocarpa *[25]. TMV-Cg virus infection in *Arabidopsis* causes the accumulation of miR160 and miR164 [54]. Three auxin responsive factor (ARF) genes (ARF10, ARF16, and ARF17) are the targets of miR160 [55]. Repression of ARF10 by miR160 is critical for the seed germination and post-germination stages [56]. MiR164 has been predicted targete six NAC-domain proteins (PNAC041, PNAC042, PNAC151, PNAC152, PNAC154, and PNAC155) from subfamily NAC-a [57], and NAC-domain proteins have been confirmed to be important in drought stress tolerance [58,59]. These mechanisms may also be at work in drought-stress tolerance in *P*. *trichocarpa* for these two miRNAs.

Two downregulated miRNAs (Ptc-miR408 and Ptc-miR1444) have been reported to be Cu-responsive miRNAs in *P*. *trichocarpa*. Their targets include miR408-targeted plastocyanin-like proteins and miR1444-targeted all plastid polyphenol oxidases [60,61]. Drought treatment may increase the relative concentration of Cu ion in the cytoplasm. When the Cu supply is sufficient, it is envisaged that the conjunction between mature miRNAs and their precursors will be suppressed, leading to the upregulation of miRNA-targeted Cu proteins [60]. Accordingly, the balance of Cu ion contributes to the healthy growth and development of poplars during stress. In *P*. *trichocarpa*, Ptc-miR1444a is reportedly downregulated by dehydration [25], and Ptc-1444b/c was also found to be downregulated by drought in this study. MiR408 is reportedly downregulated by drought stress in rice [13] and has been experimentally identified to target an early responsive dehydration-related (ERD) protein in *P*. *trichocarpa*. Drought stress might induce the expression of ERD protein by downregulating the expression of miR408 in *P*. *trichocarpa*. This may be one of the mechanisms of regulation of drought-stress tolerance [25].

Other downregulated miRNA is Ptc-miR394, whose predicted targets are annotated as dehydration-responsive protein (POPTR_0002s07760.1) and F-box proteins (POPTR_0001s13770.1 and POPTR_0003s16980.1), which were recently reported to be differentially regulated by stress conditions and to play significant roles in the abiotic stress-response pathway. In *Arabidopsis*, salt-induced miR394 targets the mRNA of F-box proteins [12,56].

From the analysis of predicted targets to downregulated Ptc-miRn6, a CCCH-type zinc finger protein and two trichome birefringence-like proteins (TBLPs) were functionally predicted. Although a cotton CCCH-type zinc finger protein has been identified to enhance abiotic stress tolerance in tobacco [62], we did not find any additional possible regulatory mechanisms between CCCH-type zinc finger protein and drought tolerance in *P*. *trichocarpa*. The homolog of TBLP in *Arabidopsis* is important to the formation of crystalline cellulose in trichomes [63]. As previous studies have reported, trichome density increases with water shortage [64], and the thick trichome layer could prevent water loss [65]. This may be the mechanism by which miRn6 regulates the expression of TBLP to adapt to drought stress.

### Degradome analysis of non-drought-responsive miRNAs

In *Arabidopsis*, miR390 was reported to target TAS genes [66], while in *P*. *trichocarpa,* no TAS homologs have been found [26]. From our study, the degradome sequencing data proved the adjustment mechanism of Ptc-miR390 and lipoxygenases (LOXs). The activity of LOX protein can partially reduce the production of radicals and ROS [67]. This may explain the regulatory mechanism of miR390 in poplars. Four UDPGs were found to be targeted by Ptc-miR482, and all were classified as category I. The UDP-glucosyltransferases (UDPGs) are enzymes that attach a sugar molecule to a specific acceptor in plants [68]. As in *Arabidopsis*, the UDPG is a key regulator of stress adaption through auxin IBA [69] and plays a role in fine-tuning nitrogen assimilation in cassava [70]. This is a novel mechanism by which miR482 regulates the UDPG gene family in *P*. *trichocarpa*.

The degradome sequencing results imply that the miRNAs with no detected targets may silence genes by repressing translation. However, we could not obtain information about translation repression by miRNA through degradome sequencing. Only 19 targets of new miRNAs were identified. The targets of these non-conserved miRNAs are difficult to detect, possibly because of low abundance or a spatial expression pattern. More studies are needed to shed light on the regulation network of these miRNAs in *P*. *trichocarpa*. Over-expressing or repressing expression of these miRNAs in *P*. *trichocarpa* may help to elucidate the regulation mechanism.

## Conclusions

In this study, sRNA libraries and degradome libraries of control and drought treatments were constructed with poplar leaves for high-throughput sequencing. Twelve miRNA members in 11 families were confirmed to be responsive to drought stress, and 65 novel miRNAs with star sequences of 59 families were identified. Through degradome sequencing, 53 and 19 genes were identified as cleavage targets of annotated miRNAs and new miRNAs, respectively. The functions of miRNA targets were analyzed and discussed. This study provides useful information for further analysis of plant miRNAs and drought stress tolerance, particularly in *Populus* plants.

## Methods

### Plant materials and total RNA extraction

*P*. *trichocarpa* seedlings of the same size (~5 cm) from tissue culture were planted in individual pots (15 L) containing loam soil and placed in a greenhouse at Beijing Forestry University. They were well irrigated and grown under control conditions (25°C day/20°C night, 16-h photoperiod) for three months, the heights of them were about 45 cm. During the period of drought-stress treatment, *P*. *trichocarpa* seedlings were sustained at two RSMC levels (70–75% and 15–20%) for 1 month according to a previous publication [28]. The mature leaves were used as drought materials. Mature leaves from soil with sufficient irrigation (RSMC at 70–75%) were used as a control, and a relatively modest dehydration level (RSMC at 15–20%) was chosen for the drought treatment. Each treatment contained three repeat individuals. Leaf water potential (WP) was measured by PsyPro WP data logger (Wescor) (Additional file 8: S9). Photosynthetic rate, water conductance, intercellular CO2 concentration, and transpiration rate were measured by Li-6400 Photosynthesis System (Li-Cor) (Additional file 9: S10). For material harvest, mature leaves from the same position of different individual plants were collected and frozen immediately in liquid nitrogen for RNA extraction. The total RNA was extracted by the standard CTAB method for plants [71]. Then they were used for sequencing and RT-qPCR.

### High-throughput sequencing and bioinformatics analysis

Illumina sequencing on sRNAs (ranged from 18 nt to 30 nt) was conducted using an Illumina Genome Analyzer, following the Illumina protocol [72]. After removing contaminants, low-quality sequences, and <18 nt sequences, clean reads were obtained and aligned against the *P*. *trichocarpa* genome (version 2.0) using SOAP software [73]. tRNA, rRNA, snRNA, snoRNA, and some other repeat sequences were removed from the sequences with a perfect match to the genome through a search of the NCBI Genbank and Rfam databases [74]. The remaining unique sequences were divided into known miRNAs and candidate miRNAs by alignment with the miRbase 18.0 [75]. The candidate miRNAs were further analyzed by MFOLD software on the RNA secondary structure of the miRNA::miRNA* and pre-miRNA hairpin energy [76]. Parameters were set to meet the criteria of plants [32].

### Differentiatial expression analysis of miRNAs between the two treatments

The sequence reads of the two libraries were normalized to 1 million by the total number of sRNA reads in each sample. The calculation of the *p*-value for comparison of the miRNA expression between the two libraries was based on previously established methods [77,78]. Specifically, the log_2_ ratio formula was: log_2_ ratio = log_2_ (miRNA reads in drought treatment/miRNA reads in control).

The following *p*-value formulae were used:

$$\begin{array}{c} p\left(x\left|y\right)\right)={\left(\frac{N_{2}}{N_{1}}\right)}^{y}\frac{\left(x+y\right)!}{x!y!{\left(1+\frac{N_{2}}{N_{1}}\right)}^{\left(x+y+1\right)}};\\ \;p=\min\left\{\sum_{k=0}^{k\leq y}p\left(k\left|x\right)\right),\sum_{k=y}^{\infty }p\left(k\left|x\right)\right)\right\}. \end{array}$$

where N_1_ is the total number of reads in the sequencing library of the control, N_2_ is the total number of reads in the sequencing library of the drought treatment, x is the number of reads for an miRNA in the control library, and y is the number of reads for an miRNA in the drought treatment library.

All calculations were performed on a BGI Bio-Cloud Computing platform (http://www.genomics.cn/en/navigation/show_navigation?nid=4143). Normalized miRNAs of <1 were filtered in both libraries.

### RT-qPCR of mature miRNAs

To validate the results of miRNAs from high-throughput sequencing, RT-qPCR was performed. The RNAs were extracted from leaves using the CTAB method [71]. A poly (A) was added to the 3’ end, and reverse transcription was begun. In particular, a known sequence at the 5’ end of the oligo-dT primer was designed to be a communal reverse primer of the RT-qPCR. The One Step Prime-Script miRNA cDNA Synthesis Kit and SYBR Premix ExTag II (TaKaRa) were used. All primers used in this study are listed in Additional file 10: S8. The 5.8S ribosomal RNA was used as the internal control [25]. RT-qPCR was performed using an ABI StepOnePlus instrument. Calculation of RT-qPCR results were revised as follow: Sample cycle threshold (Ct) values were determined and then standardized based on the 5.8S gene control primer reaction, and the 2^-ΔΔCT^ method was applied to calculate the relative changes in gene expression from RT-qPCR experiments [79].

### Target prediction and confirmation by degradome sequencing

New *P*. *trichocarpa* miRNA targets were predicted as described before [36,80-82]. During the prediction, a penalty score (alignment score) criterion was induced according to the alignment between the miRNA and its potential target. Our cut-off values in both prediction and degradome sequencing data analysis were also set to <2.5 as used in previous studies on poplar miRNA target prediction. The biological function of the predicted targets was retrieved from the Universal Protein Resource (http://www.uniprot.org).

Degradome sequencing following the PARE protocol was used [38]. Only miRNA-cleaved mRNA and other degraded mRNA could be ligated by a 5’ RNA adapter because the 5’-phosphate and intact mRNAs were protected by the 5’ cap. First, adapters and low-quality nucleotide reads were removed from raw reads using the Fastx-Toolkit. Then the clean reads were further analyzed by Cleaveland 2.0 software [83]. Briefly, the reads were first mapped to the *P*. *trichocarpa* transcripts database from JGI Phytozome 2.0. At this step, a target plot was also created to distinguish the true miRNA cleavage site from background noise. We ran Cleaveland 2.0 with default parameters using 100 randomized sequencing shuffles. The NCBI database was used to predict functions of targets that were not annotated in JGI Phytozome 2.0. The cleaved target transcripts were categorized into three categories according to the following criteria: I, the abundance of reads in its cleavage site is the maximum on the transcript; II, the abundance of reads in its cleavage site is not the maximum, but is equal to or higher than the median for the transcript; and III, the abundance of reads in its cleavage site is less than the median for the transcript.

## Abbreviations

miRNA: MicroRNAs; P. trichocarpa: *Populus trichocarpa*; sRNA: Small RNA; RISC: RNA-induced silencing complex; mRNA: Message RNA; M. truncatula: *Medicago truncatula*; P. euphratica: *Populus euphratica*; CL: Control library; DL: Drought library; siRNA: Small interfering RNA; MFE: Minimum free energy; RT-qPCR: Quantitative real time polymerase chain reaction; SOD: Superoxide dismutase; P. beijingensis: *Populus beijingensis*; MSR: Methionine sulfoxide reductase; VEP1: Vein Patterning 1; ARF: Auxin responsive factor; LOX: Lipoxygenase; UDPG: UDP-glucosyltransferase; RSMC: Relative soil moisture content; PARE: Parallel analysis of RNA ends; TBLP: Trichome birefringence-like protein; ERD: Early responsive dehydration-related.

## Competing interests

The authors declare that they have no competing interests.

## Authors’ contributions

PS DL designed and conducted the experiments, PS DL ZZ analyzed the data, PS DL WY XX drafted the manuscript, WY XX supervised the project. All authors have read and approved the final version of this manuscript.

## Supplementary Material

Additional file 1: S1 Summary of *P. trichocarpa* small RNAs sequencing. Click here for file

Additional file 2: S2 30 undetected miRNAs in this study. Click here for file

Additional file 3: S3 Potential miRNA candidates without miRNA*s found only in one library of *P. trichocarpa.* Click here for file

Additional file 4: S4 The predicted hairpin structures of all the 65 new miRNAs precursors. Click here for file

Additional file 5: S5 MiRNAs in response to drought stress. Click here for file

Additional file 6: S6 Targets prediction of new *P. trichocarpa* miRNAs. Click here for file

Additional file 7: S7 Summary of *P. trichocarpa* degradome sequencing. Click here for file

Additional file 8: S9 Leaf water potential. Click here for file

Additional file 9: S10 Leaf photosynthetic data. Click here for file

Additional file 10: S8 The primers designed for RT-qPCR. Click here for file
