# Supplementary material for: Identification of drought-responsive and novel Populus trichocarpa microRNAs by high-throughput sequencing and their targets using degradome analysis
Source: BMC Genomics. 2013 Apr 9;14:233. doi: 10.1186/1471-2164-14-233 (PMC3630063; doi:10.1186/1471-2164-14-233)
Supplement: Additional file 4: S4 — The predicted hairpin structures of all the 65 new miRNAs precursors. [file 1471-2164-14-233-S4.pdf]

Supplementary file S4- The predicted hairpin structures of all the 65 new miRNAs precursors

(The mature miRNA and miRNA* sequences are colored in red and blue, respectively.)

Ptc-miRn1 (ΔG = -56.30)

```
          10        20        30            40
-       C                   UG          .-AA|   G
 GAUGAUG CACUCCAUCAAUUCAUAG  GCACUUCAC     AUU  U
 CUAUUAC GUGAGGUAGUUAAGUAUU  CGUGAAGUG     UAA  G
A       C                   GU          \ --^   A
 .      100          90        80

                                                50
                                                 A------     AU
                                                        AAUGU  U
                                                        UUAUA  U
                                                 AAAUCGA     AG
                                                   70         60
```

Ptc-miRn2 (ΔG = -67.20)

```
       10        20         30        40            50
CA     ACC                     C            .-CG|      G
  AGGUU   AGCAACACUUGCAUUACCAAAA AGCUUCUUCAA     UUUGAA U
  UCCAA   UCGUUGUGAACGUAAUGGUUUU UUGAAGAAGUU     AAAUUU U
AC     ---                     A            \ --^      C
          170       160        150        140      60

                   70       80
             .-GUA    U   CUC   CAA
                  CCAA GCA   UGG   \
                  GGUU CGU   AUC   C
             \ ---    -   A--   UCU
                          90

                    100       110
                ------   U     GA      A
                      ACAA CUCC  UUAUAA \
                      UGUU GAGG  AAUGUU U
                CCUAGA   U     --      C
                      130         120
```

Ptc-miRn3 (ΔG = -106.50)

```
         10        20        30       40         50        60       70          80
-       A                                                      A       .-AUCUUU|   UU  UGC
 UUUUUCA UUUAGAUUGUUUUUAUGCUUUGAAUUUAUCUGUUUUCUUGGGUUGUGAUUUGUU GUGUUUG       GCAUG  CC   \
 AAAAAGU AAAUCUAACAAAAAUACGAAACUUAAAUAGGCGAAAGAACCUAACACUAAACAA CACAAAC       CGUAU  GG   U
U       C                                                      A       \ ------^   UG  UCU
 .      200        190       180       170      160        150                100        90
```

```
          110
 U--   --    AUGU  GU
   GUUU  UCUA    GUU  \
   CAAA  AGAU    CGG  A
 CCU   CU    GU--  AG
140      130
```

Ptc-miRn4 (ΔG = -66.80)

```
        10          20        30         40        50
GCAACUAAACCAA--                                      GU-|    CU
               GACUAUCCCACGAUUUAUUUGAAGAGCAAUAGCCAUUU   ACAG   \
               CUGAUAGGGUGCUAAAUAAACUUCUCGUUAUCGGUAAA   UGUU   C
AACUGUUCACUCGAC                                      AAC^    CA
120       110       100        90        80         70
```

Ptc-miRn5 (ΔG = -48.20)

```
       10        20       30        40         50
U| CG    AAGAAAAAUAGCA                                   C
 CC  AAGA            AUUACAGUGUUAAUAACAACAUUCUUCAAAAAAUA \
 GG  UUCU            UAAUGUCACAAUUAUUGUUGUAAGAAGUUUUUUAU U
-^ AU    -------------                                   G
  100                     90       80         70          60
```

Ptc-miRn6a (ΔG = -64.50)

```
         10        20        30         40         50        60
U| A      C   A                       UGUAC        GGAA  GGAA  GA
 GG UGGGAU AGC AGGGGCAAAGAUGCAUAAGAGAGA     AAGGGUAG    GG    GA  A
 CC ACUCUG UCG UCCCUGUUUUUACGUAUUCUCUUU     UUCCCAUC    CU    CU  A
-^ -      A   C                       UUC--        AG--  AUAG  AG
         120       110        100        90          80          70
```

Ptc-miRn6b (ΔG = -63.20)

```
       10       20       30       40       50        60
U| A      CA                G           UGUAC      -     AGGGGAA  GA
 GG UGGGAU  GCGAGGGGCAAAAAUG CAUAAGAGAGA     AAGGGU GUGGA      GA  A
 CC GCUCUG  CGCUCCCUGUUUUUAC GUAUUCUCUUU     UUCCCA CAUCU      CU  A
-^ -      AC                -           UUC--      U     AUAG---  AG
             120      110       100       90        80          70
```

Ptc-miRn6c (ΔG = -67.70)

```
            10        20         30         40        50        60        70
AUUC---|   A      CA                G           UGUAACAAGGGUU       GAAG   AAGA
       AUGG UGGGAU  GCGAGGGGCAAAAAUG CAUAAGAGAGA            GGGAAGGG    AGA    U
       UACC GCUCUG  CGCUCCCUGUUUUUAC GUAUUCUCUUU            UCCUUCCC    UCU    C
AUUGUUA^   C      AC                -           ------------       AUCA   AUAG
  140       130        120       110         100                     90        80
```

Ptc-miRn6d (ΔG = -51.60)

```
        10        20         30          40
U C   C   A            G            .-UG| A
 G GAC GGC AGGGACAAAAAU GCAUAAGAGAGA   UAC G
 C CUG CCG UUUUUGUUUUUA CGUAUUCUCUUU   AUG G
G U   A   C            -            \ --^ G
 120      110        100         90

                                      50       60
                                      G        AAG    AAGA
                                       GGAAGGGG   AGA     U
                                       UCUUCCCC   UCU     C
                                      U        GCA    AUAG
                                             80        70
```

Ptc-miRn7a (ΔG = -60.70)

```
        10       20         30         40        50
GG|      AG             G          UACAGU    A     AGAAAG
  GAUCGGC  GGGACAAAAAUG CAUAAGAGAGG      GGG GGCAGG     \
  CUGGCCG  CUCUGUUUUUGC GUAUUCUCUCU      CCC CCGUCU     A
CA^      CU             -          UUCUU-    A     AUAACC
 110      100           90       80         70       60
```

Ptc-miRn7b (ΔG = -61.60)

```
         10        20        30         40        50
GG|       AG             G          UACAGU   A     AGAAAG
   GAUCGGC  GAGACAAAAAUG CAUAAGAGAGG      GGG GGCAGG      \
   CUGGCCG  CUCUGUUUUUGC GUAUUCUCUCU      CCC CCGUCU      A
CA^       CU             -          UUCUU-   A     AUAACC
 110       100        90         80        70         60
```

Ptc-miRn8 (ΔG = -95.60)

```
                10         20         30          40         50          60       70          80            90      100
CUAA     -----   -                    C         G  A            .-AAA      UGG       G    A  ---| AAA    UC    UU
    CAUCC     CUC  GGGUGGCGUUUGGUUAUUGU  UCGAGACA AA AGAUGGAGACA     ACUUUUU   UUGAAGA GCAG GA   CA   UAAA  UGUC  \
    GUAGG     GAG  UCCAUCGCAAACCAAUGACA  AGCUUUGU UU UCUGCCUCUGU     UGAAGAA   AACUUUU UGUC CU   GU   AUUU  AUAG  U
A---     AACAU   U                    U         A  C            \ ---      CAA       A    -  UAA^ GAG    UA    UG
     230        220        210         200        190        180              140        130        120        110

                                                                      150
                                                                     AUAAG-------   C
                                                                                GAGA A
                                                                                UUCU U
                                                                     CACCCCCAUCUA   G
                                                                         170       160
```

Ptc-miRn9 (ΔG = -54.40)

```
       10         20         30          40          50
GCU                                AC--    A    -| UUAA     UA
  CCAUAAUCGUCUAGUGAUCGAUAAGAAAUU    CUAU UUC AU    AAAGU  \
  GGUAUUAGCAGAUCACUAGCUAUUCUUUAA    GGUA AAG UG    UUUCG  A
AGC                                GAAA    G    A^ UAAG     UU
   110        100         90         80        70        60
```

Ptc-miRn10 (ΔG = -63.40)

```
        10         20        30          40           50        60           70        80
A      G     C     -      A        .-GC   A       GC--    CGUCUCUU--     CUU-|        A AUA
 UUUGGU ACUGU UCGGG ACAGAA AGACAAAG   GGU GGAAAGA    GACA           UAUCU    GUUUUGGA GU   A
 AAGCCA UGGCA AGUCC UGUCUU UCUGUUUC   CCA UCUUUCU    CUGU           AUAGG    CAAAACCU CA   A
C      A     -     U      C        \ --   A       GCCU    AUCUUUAUUU     UUAU^        - CUU
 190       180        170       160       130       120       110        100         90

                                     140
                                    AAACA   UUCU
                                        UGU    A
                                        ACA    U
                                    UA---   CCCU
                                                   150
```

Ptc-miRn11 (ΔG = -48.60)

```
       10         20        30        40
UCUCGGA|             U                   U
       ACAAUAGUUCAAUC UUUCAGCAACCUUAAAUAG G
       UGUUAUCAAGUUAG AAAGUCGUUGGAAUUUAUC G
UGUUCAG^             C                   G
      80       70         60        50
```

Ptc-miRn12 (ΔG = -57.70)

```
        10        20        30        40          50         60        70
C     AU                        G        CAA   .-AAUAAUUAUAUU|    UAAAUUC
 CUCUC  GUUCUUGAGAACAUGAUGAAUCGU UUUAAAUU   AUC               UUAGCA      \
 GAGAG  UAAGAACUCUUGUACUACUUAGCA AAAUUUGA   UAG               AAUCGU      A
U     CU                        A        AC-   \ ------------^    UCCAUGA
     150       140       130        120                          80
```

```
                                                    90
                                                --      UCUG
                                                  UUAUAUA    \
                                                  AAUGUAU    A
                                                AA      CAAA
                                                110      100
```

Ptc-miRn13 (ΔG = -60.10)

```
         10        20        30         40
U                  A           A      -|      UU
 UGUUUAGCUUGAUGAUUA UUGACUGCAAA GCAACU CUCUUCU  U
 AUAAAUCGAACUACUAAU AACUGACGUUU CGUUGA GAGAGGA  U
C                  G           C      G^      UU
      90        80        70        60         50
```

Ptc-miRn14 (ΔG = -58.60)

```
         10         20        30
-|                                   UAA
 UUUGUUACUAAUCAUAUAGGUUGAUCCUCGUCAAUC   \
 AAACAAUGAUUAGUAUAUCCAACUAGGAGCAGUUAG   A
U^                                   CUU
.        70        60        50
```

Ptc-miRn15 (ΔG = -66.70)

```
          10        20         30         40
-                   U   A        A     GG  -    -|   UA
 GGAGGGGAGUUCUGUCGC GG AAGAUGGU CCUA  GC UGC AUAU  A
 UCUCCCCUCAAGGCAGCG CC UUCUACCA GGAU  CG ACG UAUG  U
A                   C   -        C     AA  A    A^   UG
.        90        80         70         60        50
```

Ptc-miRn16 (ΔG = -48.90)

```
        10        20         30         40       50
G|      UGUUUUUU   G          A  A        AG     A     U----   C
 AUGCCUA        UCU UCACAAUGCC AU CCUAUCU  ACCAC GGAU     UGC A
 UACGGGU        AGA AGUGUUACGG UA GGGUAGA  UGGUG UCUA     ACG A
-^      --------   -          C  C        CU     A     CACUU   U
 110               100         90         80        70         60
```

Ptc-miRn17 (ΔG = -47.30)

```
        10               20         30         40         50
CAGAG    ------|      C      U    C     U      C  A      G
     AAGGA     AGGUUU AGACGC CGC AGCGU GCACCA CA AACUU C
     UUCCU     UCUAAA UCUGCG GUG UCGCA CGUGGU GU UUGAA A
G----    CAAUUC^      A      U    -     C      A  C      A
      100        90         80          70         60
```

Ptc-miRn18 (ΔG = -64.60)

```
          10         20         30         40       50
-|                                            G     AUUU  AGG
 AUUUGAUGUGUUUGAAAUUGAACAAAUGGUAACAACCAUG CAUGG    GC   \
 UAAACUACACAAACUUUAACUUGUUUACCAUUGUUGGUAC GUAUU    UG   A
U^                                            -     ACUU  AAA
 .       100         90         80         70       60
```

Ptc-miRn19 (ΔG = -81.10)

```
         10        20        30        40         50        60          70
AUUCAAAUU                         CA        A      AC        C       .-AAUUUC| GC
         UAUUUCAAGUUCAUGCAUGCAACC  AAUUUAUA UUGUCC  ACCUGUG CAAAUUUA        CC  A
         AUAAAGUUCAAGUACGUACGUUGG  UUAAAUAU AACAGG  UGGACAC GUUUAAAU        GG  U
CGAUCUCGC                         AG        A      --        A       \ ------^ AU
        200      190       180        170        160          150                80

                                                                            90         100
                                                                      UAA--      .-AUU      CC
                                                                           GUAGAU     UUGUCU  A
                                                                           UAUCUA     AACGGA  U
                                                                      CGUUA      \ ---      CU
                                                                        140              110

                                                                                          120
                                                                                  C----  AAA
                                                                                       CA   A
                                                                                       GU   A
                                                                                  AAAAA  CAA
                                                                              130
```

Ptc-miRn20 (ΔG = -48.50)

```
         10        20        30          40          50
-                              .-CUUAAUC  A  --|    A  AG
 AUGUUGGAGUUUGAGAAAAGUCAAUCGGAC         GG CC  UUAA UG  C
 UACAACCUCAAGCUCUUUUCAGUUAGCUUG         CC GG  AAUU AC  U
U                              \ -------  A  CU^    A  GG
   110       100       90                        60

                                70
                                 U--    A
                                   UAAA \
                                   AUUU A
                                 AAC    A
                                    80
```

Ptc-miRn21 (ΔG = -46.60)

```
                10       20       30       40       50       60
AGC-----------|      CCUAUUUCA  U       A          U        C   U   A
              AGAAAGCC        UGU CAGAUC GUAGAUAGCA GUAGUUUG UUG GGUA U
              UUUUUUGG        ACA GUCUAG CAUCUAUCGU CAUCAGAC AGU CCAU G
UAUAGACCUCCCCU^      ---------  C       G          U        U   U   G
      120      110            100       90       80       70
```

Ptc-miRn22 (ΔG = -49.30)

```
        10        20         30        40            50
A    A   A                A   U      CACAG----|     CAG
 UCCA GUGG CUUCCCCUUACAAUCU CUCU UCAGUUU        AAGAUC   \
 AGGU CACC GAAGGGAAUGUUAGA GAGA AGUUAAA        UUUUAG   A
-    A   C                C   U      CAAACGGAA^     AUU
 .      100        90        80        70        60
```

Ptc-miRn23 (ΔG = -74.30)

```
         10       20         30          40         50         60
A                A  C   -           A  .-CC       A-   UAAU   .-AGA|  A
 AUUAAGUCCUCUAUCU UC ACC UUCUUCAAUC GAU    UUACGUU  GAA   UGAG     GAA  \
 UAAUUCAGGAGAUAGA AG UGG AAGAAGUUAG CUA    AAUGUAA  CUU   ACUC     CUU  A
-                C  A   A          C   \ --       CA   ----   \ ---^  A
 270       260      250        240                140               70
                                                                            80
                                                               AC-----     .-A     UU
                                                                      GUGUU   UUCAU  \
                                                                      CACAA   AAGUA  G
                                                               CAAAAAA      \ -    UU
                                                               130                90
                                                                                         100
                                                                             ACUA---------  CC
                                                                                          UC  \
                                                                                          AG  A
                                                                             AUUCCGAAAAACC  UA
                                                                           120       110
                                       150        160
                                       UGC    .-GAG    G
                                          UCAG     AACUA A
                                          AGUC     UUGAU U
                                       AGA    \ ---    C
                                          230      170
                                                     180      190      200
                                                A A    UC    AUAAUA    AAU
                                                GA AAC  UUCGA     GGUGU  A
                                                CU UUG  AAGUU     CCACA  U
                                                A G    UC    CUG---    AUU
                                                     220         210
```

Ptc-miRn24a (ΔG = -31.90)

```
         10       20        30         40
G|     A      CG    C        A      CAUUUAA
 UUUAUC CUCGAA  UUGA CGAAUGUG AUGAAA       \
 AAAUAG GAGCUU  AACU GCUUAUAC UACUUU       C
A^     G      AU    A        A      AUACUAC
       80        70         60        50
```

Ptc-miRn24b (ΔG = -35.40)

```
          10       20        30         40
-|      A      CG    C        A      C  AUAA
 GUUUAUC CUCGAA  UUGA CGAAUGUG AUGAAA AU    \
 CAAAUAG GAGCUU  AACU GCUUAUAC UACUUU UA    C
A^      G      AU    A        A      A  CUAC
        80        70         60        50
```

Ptc-miRn25 (ΔG = -35.40)

```
        10         20        30         40        50
A        C       -|         AAC     U   G  AC   AA   UGG
 AGUGAAAG UAUUUCA CUAUUCUGUG   ACAAU CUU AA  ACU  UCA   \
 UUACUUUC AUAAAGU GAUAAGACAC   UGUUA GAG UU  UGA  AGU   U
-        A       G^         A--     C   G  A-   A-   UUU
       100         90       80            70          60
```

Ptc-miRn26 (ΔG = -53.10)

```
              10        20        30         40
    G         |                                A
     GCAUAU--GCUUUACCAAGUUUCAAAUUCUCAAUAUAUACAU A
     UGUGUA  CGAAAUGGUUCAAAGUUUAAGAGUUAUAUAUGUG G
    A      \ ^                                 C
                     70        60       50


           80
            U  UA
             CUU \
             GAA A
            A  UU
             90
```

Ptc-miRn27 (ΔG = -58.00)

```
        10         20        30         40
A|   AA  A                                     A
 GUGG  UG UUUGCUAGGACCAAGUUUUUUGGAAAAUGUUAAGAUU A
 CAUU  AU AAACGAUCCUGGUUCAAAAAACCUUUUACAAUUCUAA U
-^   CG  A                                     U
       90        80         70        60         50
```

Ptc-miRn28 (ΔG = -64.00)

```
        10        20        30        40         50          60       70
UUUUUUUUA                      U          UAAU-    AA   UG .-GAGU|      AA    GGG
        AAAUGAUGACAUGGACACCAAAA CUUACAAGAA     GGUU  UGU U       GAGCAUA  AUUU    \
        UUUACUACUGUACCUGUGGUUUU GAAUGUUUUU     CCAG  AUA G       UUUGUGU  UAAG    A
AUAGGUAA                       U          UUAAU    AA   GU \ ----^      A-    UGU
    180      170       160       150       140       130             90        80
```

```
                                                             100        110
                                                           C      UAAUUG    C
                                                            UUGGGU      GUGU A
                                                            GAUCCA      UACG U
                                                           A      ------    G
                                                                120
```

Ptc-miRn29 (ΔG = -88.40)

```
        10        20         30          40
C        G       C                 .-C|    AU
 CCCCAUAA UGUGGGC UGGAGUUUCUGUGUGGC   GUGCC  U
 GGGGUAUU ACACCCG ACCUCAAAGACACACCG   UACGG  G
-        G       A                 \ -^    CG
   160       150       140             50
```

```
                                           60         70       80          90       100
                                     CAAUCGAACAACUUUAUCCUCC   A    UCC--     UCCU     U
                                                           ACA GCUG     CCAGC    UUACC  \
                                                           UGU CGAC     GGUCG    AAUGG  C
                                     AC--------------------   C    UCACA     UUU-     U
                                   130                            120       110
```

Ptc-miRn30 (ΔG = -54.50)

```
          10        20        30        40        50
-|    GG                          G   A   UGUU-    -  UUUUUGU
 UUUAA  GGGUGAUCUAGAGAACCGUUGCUUUU UUA AGU     GAGG C       \
 AAAUU  CCCACUAGAUCUCUUGGCAACGAAAA GAU UUA     CUCC G       G
G^    A-                          -   A   UCUUU    U  UUUUAAC
    110        100        90         80        70         60
```

Ptc-miRn31 (ΔG = -97.10)

```
            10       20       30       40       50       60       70       80       90      100        110      120
-------|          CAGUU            AGA              G UUCAGU           C   ACGAAC    A    AC     CU     G  C---    A     ACAAACCAU
      GGGAAGAAUACC     AGUUACAUGUAG   CCACCAAACCCAAA U      UCACUUUCUGA AGA      CCUA CUCA  CUUUU  CACUC AG    AGUC CAGGG         \
      UCCUUCUUAUGG     UUAAUGUACAUC   GGUGGUUUGGGUUU A      AGUGGAAGAUU UCU      GGAU GAGU  GAAAA  GUGAG UU    UCGG GUUCC         C
CCGUCGC^          -----            ACC              G ------           C   CA----    A    --     UG     G  AGCU    -     CAAAAACUU
       230      220           210      200      190           180      170         160        150      140       130
```

Ptc-miRn32 (ΔG = -67.80)

```
        10         20        30          40       50         60       70
G     U   U          A      GC     .-AACCAUGGUCAUAUC|       U  UAU      UA
 GGGGU UCU GUCGCAGGAG GAUGGC  UAGCU                  AUAUAUA CA   GGCAAG  \
 UCCCA AGG CAGCGUCCUC CUACCG  AUCGA                  UAUGUAU GU   UCGUUC  U
G     C   U          -      AA     \ ---------------^       -  UUC      AU
 140        130         120                           90         80


                                         100
                                    C      A
                                     AGCUGU A
                                     UCGAUA A
                                    C      G
                                   110
```

Ptc-miRn33 (ΔG = -63.80)

```
        10        20        30        40
AUGUCA|                                 UGG  UG
      AGGUUAGUUCCCAACCUACACCACAAUAUGUGUG   UAU  U
      UCCAAUCAAGGGUUGGAUGUGGUGUUAUACACAC   AUG  G
UUCUAA^                                 UGG  UG
       90        80        70        60        50
```

Ptc-miRn34 (ΔG = -38.60)

```
          10        20        30        40
-    -| G                       CAAACUAG     C
 UUCC UC UCUUAAUUAGAACUCAUACUAGA        UGGAU C
 AAGG GG AGAAUUAAUCUUGAGUAUGAUCU        AUCUA U
A    U^ -                       CCUA----     U
       80        70        60            50
```

Ptc-miRn35 (ΔG = -49.00)

```
        10        20        30        40         50
C         UC   A       U-    C          AAGA-| UU      UCUCU
 GAGGAAGUU  UGG CAUGGGU  GGUU GCAAGAAAAU     UU  UGAAC      \
 CUCCUUUAG  ACC GUACCCA  CCAG CGUUCUUUUG     AG  ACUUG      G
-         UA   -       CC    C          ACAAA^ GU      UUAUA
     110        100        90        80        70        60
```

Ptc-miRn36 (ΔG = -60.30)

```
          10        20        30        40       50        60        70        80
CGGCA--     -|   U    U            C         UUAU   U       GGUU   UUAAG  AGC      G
       ACCAU UUAG CAUG UUGCUAACAUGA CGUCCAAG    CAA UAUGUCA     ACA      GA   UGGCAU A
       UGGUG AGUU GUAC AACGGUUGUACU GUAGGUUC    GUU GUACAGU     UGU      CU   ACCGUA G
UACUAGA     U^   -    C            A         UACU   U       ----   UGAAA  ---      C
      150        140        130       120        110      100             90
```

Ptc-miRn37 (ΔG = -75.80)

```
        10        20        30        40
U|                                            U
 AUAAUGCCAACCAUAGCCUCCAUUAUCACACUAUUUUGUUUGGUA \
 UAUUACGGUUGGUAUCGGAGGUAAUAGUGUGGUAAAACAAACCAU U
U^                                            U
     90        80         70         60        50
```

Ptc-miRn38 (ΔG = -44.00)

```
        10        20         30        40
AG           U                      UCU--|  CUA
  CCAAAAGUUAA AAAAUCUCGACUAUUAUCUAAAA      CGA   C
  GGUUUUUAAUU UUUUAGAGCUGAUAAUAGAUUUU      GCU   U
AG           U                      UUUUU^  UCU
    90         80        70        60         50
```

Ptc-miRn39 (ΔG = -41.10)

```
         10        20        30        40
GAG     -|  G      U      CU   U   UAAU    UUAC
   AAGCU UCC AUCUCCC CAAAGG  UCC CUA   UGCC     \
   UUCGA AGG UAGAGGG GUUUCC  AGG GAU   ACGG     A
UCA     G^  -      -      UC   U   U---    UAGU
90       80           70        60           50
```

Ptc-miRn40 (ΔG = -45.40)

```
          10         20        30         40         50
CCAU--|    AGU     U   G       A            U     AACAAGG
      UGCAG   AGAAG CGA CUCAAGC CAAAUUCGAUCU GCUU       \
      ACGUU   UCUUC GUU GAGUUCG GUUUAAGCUAGA UGAA       U
AAUCUU^    GCU     U   A       G            C     ACGGCCA
 .      100         90        80         70        60
```

Ptc-miRn41 (ΔG = -44.70)

```
        10          20        30          40
AG      C -       U -              -| ACAUA     UU
  AAGCUC C AACUCCC C AAAGGUUUCCAGCA UC     AUUGC  \
  UUCGAG G UUGGGGG G UUUCCAAAGGUUGU AG     UAAUG  C
CA      A U       U U              A^ GG---     AA
     90        80        70         60           50
```

Ptc-miRn42 (ΔG = -38.90)

```
        10       20         30                40
A        UG G         U  -    C      AU-------| UC
 CUGGAAGU  U GGCAUGAGG GU UUGG AAGAAA        GGA   \
 GACCUUUA  A CCGUACUCU CA AACC UUCUUU        CCU   U
-        GU A         C  U    A      AGUAGUAUU^ UU
    90        80        70         60        50
```

Ptc-miRn43 (ΔG = -52.30)

```
         10        20         30         40         50
-  CA-|        UU            A         UU   U  -   C
 CC   AAAUCGCUU  CGAAGAAUCUCA CCAUUGGAU  GUG GA CCC G
 GG   UUUAGUGAA  GCUUUUUAGGGU GGUAACUUA  CAC CU GGG G
A  AGC^        GG            A         CU   -  A   U
100        90         80          70         60
```

Ptc-miRn44 (ΔG = -55.80)

```
         10        20         30         40
- A  CA    UU                  CA-|         GGCU
 GA CC  AAUG  UUCCCUGAAUCACUCCCAC   UUGAUUGGUG    \
 CU GG  UUAC  AAGGGACUUAGUGAGGGUG   AAUUAACCAC    C
A C  AC    CC                  ACA^         GUAC
        90         80         70         60         50
```

Ptc-miRn45a (ΔG = -79.90)

```
       10       20       30       40          50       60       70       80
-    U                          AGC         ---  G      -|     AAGCACAUACCACAUUUUCU      AAAA
 AGGG GUGUUUGGGUGGGAGGUGUGGU   UUUUUUUUUA   AG UACCAC CCUCUUA                   AAAAGCA    \
 UCUC CACAAACCCACCCUCCACACCA   AAAAAAAAGU   UC AUGGUG GGAGAAU                   UUUUCGU    A
A    C                          AC-         UUU   G      U^     GAU-----------------      ACAU
160     150      140       130      120      110      100                      90
```

Ptc-miRn45b (ΔG = -78.70)

```
         10         20         30         40
-    A                          A C         .-AA| UA
 UGGG GUGUUUGGGUGGGAGGUGUGGU G UUUUUUUUU   GG  \
 GCUC CACAAACCCACCCUCCACACCA C AAAAAAAAA   CC  C
A    C                          A A         \ --^ AC
    160        150        140      130     50
```

```
                                                   60        70            80
                                            C  UU      ACAC            ---        -   AAAA
                                             UC  AAAGC     ACCACACUUU    CUAAAA GCA     \
                                             AG  UUUCG     UGGUGUGGAG    GAUUUU CGU     A
                                            A  UU      A---            AAU        U   ACAU
                                                120           110        100         90
```

Ptc-miRn46 (ΔG = -86.80)

```
        10         20       30        40        50         60         70        80
AUAUG                  A      G  A     UG A UG    UUGU   .-ACAA      .-AUUUUAGAAAUAUA|    A
     AGGAAUUCCUCCUACCCA CACGUG CA UCUCU  G C  UCAU    UGG      CAUUUGUU               UUUG U
     UCCUUAAGGAGGAUGGGU GUGUAC GU AGAGA  U G  GGUA    ACC      GUAAACAA               AAAC A
CAUCA                  C      A  C     GU G GU    CGU-   \ ----      \ --------------^    U
 250       240       230        220       210       200                              90

                                                                         100       110        120
                                                                      AUCAUACA   -     AAAAAG      UU
                                                                              UGU AUAAU      GGUAAG  U
                                                                              ACG UAUUA      CCAUUU  C
                                                                      A-------   G     GAAAAA      GU
                                                                  150      140      130

                                                           160
                                                           .-G      U
                                                              UUUUUG U
                                                              AAAAAC G
                                                           \ -      A
                                                                  170

                                                             180
                                                            AUAAAA    AU
                                                                  AGCA  \
                                                                  UCGU  U
                                                            CA----    AA
                                                                     190
```

Ptc-miRn47 (ΔG = -34.70)

```
           10         20         30         40          50         60
AAUA    --    A       U    U  U      U       --|       A    UCAUC
    AGUA  AAACA UAUGUG GAU  CC UCCAAU CCAUUUG  CUUAGUUU UUU      G
    UCAU  UUUGU AUGUAC CUA  GG AGGUUA GGUGAAC  GAAUUAAA AAA      U
GUG-    UU    A       C    U  -      C       AA^       G    UUCCC
120        110        100          90         80         70
```

Ptc-miRn48 (ΔG = -35.00)

```
       10       20       30       40
A|     ACAAU      C            GA    U  C  U
 AGAAG     CUCAAGC CAAAUUCGAUCU  CUU GC GG \
 UCUUC     GAGUUCG GUUUAAGCUAGA  GAA UG UC A
A^     AGCUC      U            AC    U  U  C
          80       70       60       50
```

Ptc-miRn49 (ΔG = -63.30)

```
          10         20         30         40         50         60         70        80          90         100        110
-    A   CUC                          A   CA    AAGC-   UC          GAC   C   CAA  GA-      .-AAAUGAAA| C  G   CCAU
 UUAU GUG   AAGGAGUAAUUAGUGACAUCUUAUUA UUA  UCCU     UCA  UUGAAGAAAGA   AGA ACA   AG   AAGAGA          CU AU GC    \
 AAUA CAC   UUCUUCAUUGAUCACUGUAGAAUAAU GAU  AGGA     GGU  AAUUUUUUUUU   UCU UGU   UC   UUCUCU          GA UA CG    C
A    C   AAC                          C   C-    AACUA   GA          A--   A   ---  AUG      \ --------^ U  G   AAAA
 .      220       210         200        190        180       170          160          150                  120

                                                                                                   130
                                                                                              AAUAACC  AC
                                                                                                     CU   \
                                                                                                     GA   C
                                                                                              CUAUA--  AU
                                                                                                  140
```

Ptc-miRn50 (ΔG = -77.10)

```
        10        20        30        40        50
U|                                      U       A
 UCCAGCAUUCUCGAUUGAAUGCCACAUAUCAUUCAUUCC GCUUUUU U
 AGGUCGUAAGAGCUAACUUACGGUGUAUAGUAAGUAAGG UGAAAAG A
-^                                      C       U
          90       80         70         60
```

Ptc-miRn51a (ΔG = -141.50)

```
         10        20        30        40        50        60         70                 80       90         100       110
-                    C   GU    UGA   G   -     CGA    U        U   C     -    --------    A     CCC   .-AUGAUUGUCCCC|    UUC
 GCUCUCCUUGCAAUCCCCGA CUC  GGCG   UGC CCC GCUGC   GGCC GGCCUCCG CUU CGAGC CGGG        GCCG CAGCC   CGC              GUCGU   C
 CGAGAGGAGCGUUAGGGGCU GAG  CCGC   GCG GGG CGGCG   CCGG CCGGAGGU GAA GCUCG GCCC        UGGC GUCGG   GCG              CGGCG   C
U                    C   --    CAG   -   U     CAG    U        U   C     U    GUCGCCGG    -     CCU   \ ------------^    CCC
    240       230        220         210      200        190      180       170       160        150              120

                                                                                                               130
                                                                                                      CU---   C  UGU
                                                                                                           GUC CU   C
                                                                                                           UAG GA   C
                                                                                                      CUUCC   A  UUC
                                                                                                             140
```

Ptc-miRn51b (ΔG = -143.00)

```
         10         20        30        40        50       60         70           80       90
-                    C   GU     UGA   G  C     CGA   U        U   C     -    .-GCU    CCCC    ---------|  UGU
 GCUCUCCUUGCAAUCCCCGA CUC  GGCG   UGU CC GCUGC  GGCC GGCCUCCG CUU CGAGC CGGG    GACAG   CCGC        AUGAU   \
 CGAGAGGAGCGUUAGGGGCU GAG  CCGC   GCG GG CGGCG  UCGG CCGGAGGC GAA GCUCG GCCC    CUGUC   GGCG        UGCUG   C
U                    C   --     CAG   G  U     CAG   U        U   C     U    \ ---    C---    CCCCCCCUU^  CCC
    240       230        220        210       200        190      180                     120      110
```

```
                                                                                130
                                                                             .-G    UC
                                                                                CUUG  C
                                                                                GAGC  C
                                                                             \ -    UU
                                                                               140
```

```
                                                                                    150
                                                                              AUCCUUC   U     U
                                                                                     GCG CCGCC \
                                                                                     CGC GGUGG G
                                                                              GU-----   C     C
                                                                             170            160
```

Ptc-miRn51c (ΔG = -137.40)

```
         10        20         30        40         50         60        70                  80        90          100      110
-                     C   GU     UGA    G  C      CGAG    U         U   C      -     --------|   A      CCC   .-AUGAUUGUCCCC     UUC
 GCUCUCCUUGCAAUCCCCGA CUC  GGCG    UGU CC GCUGC    GCC GGCCUCCG CUU CGAGC CGGG        GCUG CAGCC    CGC               GUCGU   C
 CGAGAGGAGCGUUAGGGGCU GAG  CCGC    GCG GG CGGCG    CGG CCGGAGGC GAA GCUCG GCCC        UGGC GUCGG    GCG               CGGCG   C
U                     C   --     CAG    G  U      CAGG    U         U   C      U     GUCGCCGG^   -      CCU   \ ------------     CCC
    240       230         220         210       200        190       180       170       160         150              120

                                                                                                            130
                                                                                                      CUGUCC-    UC
                                                                                                             CUUG  C
                                                                                                             GAGC  C
                                                                                                      CUUCCUA    UU
                                                                                                             140
```

Ptc-miRn51d (ΔG = -137.60)

```
         10         20        30        40         50        60         70          80        90
-                     C    GU     UGA   G  C      CGA     U        U    C      -    .-GCU    CCCC    --------|    UGU
 GCUCUCCUUGCAAUCCCCGA CUC   GGCG    UGU CC GCUGC   GGCC GGCCUCCG CUU CGAGC CGGG    GACAG    CCGC          AUGAU   \
 CGAGAGGAGCGUUAGGGGCU GAG   CCGC    GCG GG CGGCG   CCGG CCGGAGGU GAA GCUCG GCCC    CUGUC    GGCG          UGCUG   C
U                     C    --     CAG   G  U      CAG     U        U    C      U   \ ---     C---    CCCCCCUU^    CCC
    240       230          220        210       200        190       180                     120       110

                                                                              130       140      150
                                                                             CCUU     UUC     CCUUC  G
                                                                                GUCCC   GAGAU     GC  U
                                                                                CGGGG   CUUUG     CG  C
                                                                             GC--     ---     UUC--  C
                                                                             170           160
```

Ptc-miRn51e (ΔG = -139.30)

```
         10         20       30       40       50         60        70
-                    C   GU    UGA  G  C     CGA    U        U   C    .-C|    UG
 GCUCUCCUUGCAAUCCCCGA CUC  GGCG  UGU CC GCUGC   GGCC GGCCUCCG CUU CGAGC  GGGGC   \
 CGAGAGGAGCGUUAGGGGCU GAG  CCGC  GCG GG CGGCG   CCGG CCGGAGGU GAA GCUCG  CCCCG   A
U                    C   --    CAG  G  U     CAG    U        U   C    \ -^    AC
    240       230         220       210      200       190       180
```

```
   90
CC--  .-AU  UGU
    GC   GAU   \
    CG   CUG   C
UGCC  \ --  CCC
             100
```

```
  110        120       130
 GUUUCCCCCC      .-UGUCC   UC
           GCGGCC      CUUG  C
           CGCCGG      GAGC  C
 U---------      \ -----   UU
170                    140
```

```
      150
AUCCU     UC
     UCGCG  C
     GGCGU  G
U----     CG
      160
```

Ptc-miRn52 (ΔG = -74.60)

```
         10         20         30       40        50        60          70
-    UC      UAAG -               A                 A     ACCGAG-  UU-|    U  AGG
 AAGC  CAUCCA    G UUUUAUGUUUGACAG AACCCCCUCAUGUUUUC ACCCU       GU    UUUG CA   \
 UUCG  GUAGGU    C AAAAUACGAACUGUC UUGGGGGAGUACAAAGG UGGGA       UA    AAAC GU   U
G    UA      UUA- G               G                 A     CAUGAUA  UUU^    U  CUU
     150        140      130        120      110        100       90         80
```

Ptc-miRn53 (ΔG = -42.60)

```
         10        20         30        40               50
-                A     G-    U       CAUA  U   -------|     UCAA
 GGAAAUUGAUUAAAUC AGCCC  GUAC UUUAAUU    UG GAC       CCCGUA     \
 CCUUUAAUUAAUUUGG UCGGG  CAUG AAGUUGA    AC CUG       GGGUAU     A
A                -     AA    -       A---  -   AAUAGUU^     UUGA
  110       100         90         80            70        60
```

Ptc-miRn54a (ΔG = -61.00)

```
         10              20        30         40          50        60
UUUU     -  -----      U          U             C--|         U      U
    CUCUC UG     GGCACG UGUUCAUUCC CUUCCUAAAAUGG   UUCUUCUCCU UAGUUG \
    GGGAG AU     CUGUGU AUAAGUAAGG GAAGGAUUUUACC   GAGGAGAGGA AUUAAC U
----     C  UUUGG      C          -             UAA^         U      G
         120       110       100         90        80        70
```

Ptc-miRn54b (ΔG = -50.90)

```
          10          20         30         40          50
U   -   UU  --       U                   C-    .-A|    AAA
 GCC ACG  GU  UCAUUCC CUUCCUAAAAUGGCUUCUC  UUCA   UUUUA   \
 UGG UGU  CA  AGUAAGG GAAGGAUUUUAUCGAAGAG  AAGU   AAAAU   A
G   C   GG  UA       -                   UA    \ -^    AAA
   120       110         100         90            60

                                                        70
                                                  AUUAAA  AG
                                                        GG  \
                                                        CC  A
                                                  AUAA--  UA
                                                  80
```
